# Feminist Identity and Online Activism in Four Countries From 2019 to 2023

**DOI:** 10.1177/08944393241301050

**Published:** 2024-11-15

**Authors:** Shelley Boulianne, Katharina Heger, Nicole Houle, Delphine Brown

**Affiliations:** 1 7423 University of Southampton, UK; 2 648518 Weizenbaum Institute for the Networked Society, Germany; 3 3151 MacEwan University, Canada

**Keywords:** gender, political participation, online participation, pandemic, petitions, cross-national

## Abstract

The COVID-19 pandemic heightened burdens on caregivers, but also the visibility of caregiving inequalities. These grievances may activate a feminist identity which in turn leads to greater civic and political participation. During a pandemic, online forms of participation are particularly attractive as they require less effort than offline forms of participation and pose less health risks compared to collective forms of offline activism. Using survey data from four countries (Canada, France, the United States, and the United Kingdom) collected in 2019 (prior to the pandemic), 2021 (during the pandemic), and 2023 (post-pandemic), we examine the relationship between self-identifying as a feminist and signing online petitions (*n* = 18,362). Our multivariate analyses show that having a feminist identity is positively related to signing online petitions. We consider the differential effects of this identity on participation for men, women, non-binary people; caregivers versus non-caregivers; and respondents in different countries with varying levels of restrictions due to the pandemic. A feminist identity is more important for mobilizing caregivers than non-caregivers, whether or not the caregiver is a man or a woman. While grievance theory suggests differential effects by country and time period, we find a consistent role of feminist identity in predicting the signing of online petitions across time and across countries. These findings offer insights into how different groups in varying contexts are mobilized to participate.

## Introduction

The COVID-19 pandemic has been a substantial setback in the fight for gender equality. Across the globe, daycares, schools, and group living accommodations for seniors closed down, moving care into homes where women tend to perform the large majority of care work (Calarco et al., 2021). For example, a Statistics Canada survey conducted three months into the pandemic found that women were “ten times more likely than men to say childcare fell mostly on them” (Haney & Barber, 2022, p. 26) and parents were especially likely to report that the pandemic made the division of labor worse. In this context, we might see lower levels of political participation among women or those who take on caregiver roles (Burciu & Hutter, 2022) as their free time is significantly reduced by the greater caregiving responsibilities; free time is associated with political participation (Li et al., 2023). However, this heightened injustice related to the unequal division of labor may contribute to the salience of gender and a feminist identity and may also politically motivate citizens to collectively seek institutional support (e.g., Ahn et al., 2021). More specifically, having a feminist identity may spur such efforts, including efforts to pressure political officials for solutions. In this paper, we examine two key questions. Is a feminist identity linked to greater online political participation? And, is this collective identity equally mobilizing across a variety of populations and time periods?

Using four-country (Canada, France, the United States, and the United Kingdom) survey data collected in 2019 (prior to the pandemic), 2021 (during the pandemic), and 2023 (post-pandemic), we examine the relationship between self-identifying as a feminist and signing online petitions. Due to the pandemic, online political participation is important given the restrictions on in-person contact (Ohme et al., 2021). Indeed, offline collective action may be legally inaccessible to citizens throughout the pandemic. We move beyond the simple dichotomy of women versus men’s participation, instead focusing on the impact of having a feminist identity. We view feminist identity as a collective identity tied to grievances and we consider whether this identity has different mobilizing effects for different subgroups based on the degree of grievances related to inequality tied to the pandemic. We find that feminist identity is more important for mobilizing caregivers than non-caregivers, whether or not the caregivers are men or women. In other words, feminist identity is not exclusively important for mobilizing women caregivers but is an important mobilization force for those who engage in care work.

This paper offers empirical evidence to support expanding the definition of feminism to reflect a diverse set of experiences, rather than focusing exclusively on women’s agency. In particular, we look at the role of feminist identity among caregivers and non-caregivers as well as men, women, and non-binary people, capitalizing on our large sample size which offers opportunities for more nuanced analysis. Furthermore, our results track changes in feminist identity and online political participation over three points in time (2019, 2021, 2023). The findings offer insights into the long-term effects of the pandemic as well as differential experiences of the pandemic among different subgroups (men, women, non-binary people; caregivers vs. non-caregivers) and in different countries (Canada, France, the United States, and the United Kingdom).

Beyond offering empirical evidence, this paper offers several theoretical contributions, including (1) understanding how collective identity combined with grievance theory relates to activism, (2) offering new insights into the gendering of civic and political participation, and (3) theorizing both cross-national and longitudinal differences to offer a nuanced analysis of the role of context in political participation.

## Gender and Political Participation

While some research finds that there is a gender gap in political participation, others find no gender gap, or just differences in the ways that men and women participate (Bode, 2017; Coffé & Bolzendahl, 2010, 2017; Pfanzelt & Spies, 2019). Specifically, men participate more than women in conventional and public forms, whereas women participate more in flexible, private, and individual forms of political participation (Coffé & Bolzendahl, 2010; Pfanzelt & Spies, 2019).

Other research shows that there are gender differences in perceptions of citizenship, with women viewing political, civil, and social rights as more important than men (Bolzendahl & Coffé, 2009). Focusing on rights may lead to different forms of participation (i.e., signing petitions) than focusing on responsibilities (i.e., voting). Indeed, some research shows that women sign petitions at higher rates than men (Brundidge et al., 2013; Coffé & Bolzendahl, 2010; Crepaz et al., 2016; Dodson, 2015, 2016; Elliott & Earl, 2018; Grasso, 2016; Ha et al., 2013; Pfanzelt & Spies, 2019). All of these studies affirm that to understand gender differences, we need to isolate the type of activity rather than pool them into a multi-item index or scale of political participation. When different activities are combined, the gender differences in types of activities may cancel out each other, leading to the false conclusion that there are no gender differences (Feezell, 2016; Oser et al., 2013; Wei, 2012). As such, it is important to distinguish the specific activity to understand whether gender plays a role, which will also help untangle which theoretical explanation (resource, social cognitive theory) best explains the findings.

Several factors have been used to try to explain gender differences in political participation. Socioeconomic resources such as money, time, and employment are important when considering political participation (Beauregard, 2016; Carreras, 2018; Coffé & Bolzendahl, 2010; Elliott & Earl, 2018; Páez-Bernal & Kittilson, 2022; Swank & Fahs, 2017). Indeed, free time is associated with a higher likelihood of electoral participation (Li et al., 2023). Li et al. (2023) show that working long or unsociable hours lowered women’s electoral participation but only lowered the electoral participation of men with the lowest occupational status. They suggest that this gender difference can be attributed to women’s double shifts in paid and unpaid work time.

Political efficacy and political interest are important factors in political participation (Carreras, 2018; Coffé & Bolzendahl, 2010, 2017; Dodson, 2015, 2016; Elliott & Earl, 2018; Giugni & Grasso, 2019; Heger & Hoffmann, 2021; Klandermans, 2004; Koc-Michalska et al., 2021; Oser et al., 2022; Páez-Bernal & Kittilson, 2022; Pfanzelt & Spies, 2019). Women tend to report lower levels of political efficacy and political interest than men (Carreras, 2018; Coffé & Bolzendahl, 2010, 2017; Heger & Hoffmann, 2021; Pfanzelt & Spies, 2019). Coffé and Bolzendahl (2010) find that when these factors are controlled for, gaps in many private-type political activities disappear or are reversed. Unlike Coffé and Bolzendahl (2010), Carreras (2018) did not account for political efficacy and interest, which may explain the large gender differences observed across the various political activities using data from multiple large-scale international surveys (also see Páez-Bernal & Kittilson, 2022).

Regarding online political participation, several studies show that women are less likely to engage in online political expression or posting political content to social media sites (Abendschön & García-Albacete, 2021; Bode, 2017; Boulianne, 2023; Heger & Hoffmann, 2021; Koc-Michalska et al., 2021). These studies tend to explain gender differences in socialization experiences, but also in relation to different levels of political efficacy (Heger & Hoffmann, 2021) or resources (Boulianne, 2023).

In a study of US citizens, Brundidge et al. (2013) conclude that women are shifting their participation from offline to online, which is expected to narrow online gender gaps. This statement seems to hold true even in the context of the COVID-19 pandemic. A German study showed that throughout the pandemic, women’s political participation decreased in all forms except online, where their participation increased more than men’s did (Burciu & Hutter, 2022). However, the role of gender in online and offline participation is not consistent across countries. Almost all these studies (except Boulianne, 2023; Koc-Michalska et al., 2021) are based on single-country experiences and, yet, different countries have different institutional and cultural norms that shape gender experiences (Dodson, 2015).

## Feminist Identity and Political Participation

Feminism has been defined as a belief system or ideology with a political goal, which can involve the “[elimination of] all forms of sexism and violence against women” (Evans & Chamberlain, 2015, p. 398), or simply the goal of ending sexist oppression (hooks, 1984). However, the more specific aims or goals of feminism have fluctuated over time (Lorber, 1997; McAfee & Howard, 2023). A feminist identity is conceptually and empirically different from feminist attitudes in a number of ways (Rhodebeck, 1996), even though both cognitions are deeply related and correlated (Bargad & Hyde, 1991; Heger & Hoffmann, 2022).

While much research has been done on studying men’s and women’s political participation, less is known about the effect that having a feminist identity exerts on one’s online political participation. In the offline realm, several authors find evidence for an empowering effect of a feminist consciousness on political activism (Duncan, 1999; Swank, 2021; Swank & Fahs, 2014, 2017). Szymanski (2004) confirms this relationship using a more direct measure of feminist self-identification (also referred to as feminist self-labeling). Likewise, Heger and Hoffmann (2021; 2022) find that for women, having a feminist identity increases the likelihood of online political participation and that feminism is linked to political efficacy.

In line with Social Identity Theory (Tajfel, 1978), we ground our study of feminist identity and online activism by defining feminist identity as a collective identity. Collective identity can be understood as a fluid community that emphasizes collective approaches to gender(ed) conflicts (Cowan et al., 1992; Hunt & Benford, 2004; Polletta & Jasper, 2001; Snow, 2001) and previous research has suggested that political engagement can be encouraged by collective identities (Deckman et al., 2020; Kelly & Kelly, 1994; Lacombe et al., 2019; Schwartz, 2021; Swank & Fahs, 2014; Veenstra & Haslam, 2000). Our first hypothesis is:

H1 Feminist identity positively relates to signing online petitions.

## Differential Effects of Feminist Identity: Gender, Caregivers, Country, and Time Period

Understanding feminist identity as a collective identity relates to insights from grievance theory. Following grievance theory, those who are most dissatisfied should be the most politically active as social movements can be the vehicle for the collective to voice their grievances (Ejrnæs & Harrebye, 2021; Klandermans, 2004). Essentially, being part of a stigmatized group contributes to activism on behalf of the group. Prior research supports grievance theory in that satisfied people are less likely to participate in political activities than dissatisfied people (Carreras, 2018; Ejrnæs & Harrebye, 2021; Grasso et al., 2019; Moseley, 2018). For example, members of marginalized groups such as gays and lesbians are more likely to join political movements pertaining to lesbian, gay, and bisexual rights than heterosexuals (Swank & Fahs, 2011). As such, we might expect those who experience, or are aware of structural grievances and inequality, are more likely to identify as feminists and become more politically engaged online. At a collective level, we expect that women will experience this grievance more so than men given historical differences in care work (Bianchi et al., 2000, 2012; Haney & Barber, 2022) as well as the role of the pandemic in intensifying these differences in care work. From this perspective, we propose:

H2 Feminist identity has a stronger relationship with signing online petitions among women than men.

However, feminism recognizes that the formation of a strong collectivity does not necessitate uniform identification among members (Evans & Chamberlain, 2015). Early feminism tends to be critiqued for its focus on the experiences of White, middle-class women (hooks, 1984; Spelman, 1990). A feminist identity is not exclusive to women. In a survey of US adults conducted in 2007 and 2009, Kelly and Gauchat (2016) found that both men and women identified as being feminists; the same was observed in Germany (Heger & Hoffmann, 2021).

Burciu and Hutter (2022) conducted a study in Germany about how the pandemic influenced people’s *self-reports of changes* in levels of civic and political participation. They find that while women’s political participation during the pandemic did not seem to be affected by additional care-work obligations, men with childcare responsibilities showed a stronger upward trend in political participation than childless men. They suggest that the increase in men’s care responsibilities acted as a grievance on which to act in institutional, online, and protest participation. However, these trends were not found in civic engagement (Burciu & Hutter, 2022). As such, we further examine variations based on caregiver roles. We use grievance theory to explain that caregivers will be mobilized more than non-caregivers in the context of the pandemic which highlighted inequalities in care work. Following this literature, we propose:

H3 Feminist identity has a stronger relationship with signing online petitions for caregivers (parents of children under the age of 18 years) compared to non-caregivers.

As noted above, understanding gender differences in patterns of online and offline participation requires consideration of the country context. Feminist identity may also differ in its role in different countries. Dodson (2015) finds that women participate in politics less than men in societies with traditional gender ideology environments, but in more egalitarian societies, women are more likely to participate than men. When people are asked about being a feminist, Swedish people have the highest rates of feminist identity (with French people a close second), UK respondents are somewhere in the middle in terms of rates, and Germans have the lowest rates of feminist identity (Abraham, 2018). In these different contexts, we might expect the role of feminist identity to differ in predicting patterns of political participation. Our first research question is:

**RQ1:** To what extent does the role of feminist identity on signing online petitions differ by country?

The COVID-19 pandemic is a “critical event” that can influence the opportunities to advance a social cause (Ramos, 2008). We argue that this event made grievances more salient for those seeking gender equality. COVID-19 support policies differ by country. According to data collected by the University of Oxford (Hale et al., 2021), in February 2021, all four countries had income supports in place. During the month of February 2021, Canada and the UK had more school closures compared to the United States and France. Finally, using a comprehensive measure of “stringency” for the first year of the pandemic (Mathieu et al., 2020), which includes school and workplace closures, the UK experienced greater restrictions than the other countries. Building on grievance theory, we might expect that those in the UK were suffering from greater restrictions, which may fuel demands for income and other supports. However, as the social movement scholarship has shown, grievances do not necessarily translate into political action. A variety of other factors are important to consider, including opportunity structures, collective identity, and political efficacy. Our second research question is:

**RQ2:** To what extent does the role of feminist identity on signing online petitions differ by year?

## Methods

### Sample

The survey was administered by Kantar who use a weighting efficiency measure to report on sample quality. The weighting efficiencies reflect a match between the sample characteristics and census data for each country. Because of this close match (97–99%), we did not weight the data. The surveys were conducted from September to November 2019, February 2021, and January 2023. The survey is longitudinal but not a repeated wave panel study; there were different respondents each year. The total sample size is 18,362. The year of data collection is included in the models as a linear variable, then examined with more nuance in subsequent models. There are approximately 4500 respondents in each of the countries (1500 per year). The survey was conducted in both English and French with Canadians being offered a choice of language. The data and replication files are posted to: 10.6084/m9.figshare.26672923. The surveys were funded through research grants to the first author from Canadian Heritage (Digital Citizenship Initiative) and the Social Sciences and Humanities Research Council (435-2019-04–94).

### Signed Online Petition

We asked respondents how frequently they had signed an online petition in the past 12 months. The question is asked as part of a series of questions about political participation, including voting, meeting offline, strikes, marching, volunteering for/donating to parties and/or NGOs, contacting officials online/offline, and talking politics online/offline. Four response options were given: never (1), rarely (2), from time to time (3), and often (4). The average of the pooled sample is 1.84 and the standard deviation is 1.00.

### Feminist Identity

Feminist identity can be captured in a number of ways, as identified in a systematic review of literature (Siegel & Calogero, 2021). A 2018 YouGov survey in seven European countries shows that asking if people are feminist, without offering a definition, yields the lowest percentages of people identifying as feminist. However, when provided with a definition, “a feminist is someone who thinks men and women should have equal rights and status in society, and be treated equally in every way” (Abraham, 2018, p. 2), the percentage of people identifying as feminists increases. Finally, asking, “do you think men and women should or should not have equal rights and status in society and be treated equally in every way” (Abraham, 2018, p. (2)) produced the highest levels of agreement.

**Figure 1.**
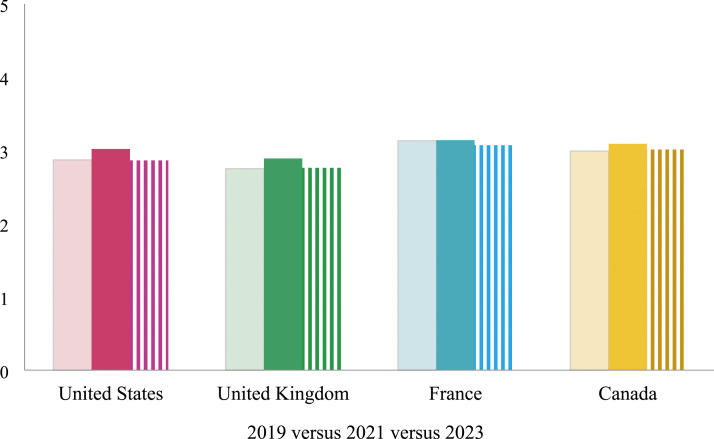
Feminist identity by year and country.

To measure feminist identity, we included the following definition and question: “Webster’s dictionary defines feminism as the belief in the political, economic, and social equality of the sexes. Based on this definition, to what degree do you self-identify as a feminist?” This approach is simple (vs. Heger & Hoffmann, 2021; 2022; Siegel & Calogero, 2021), but it is directly informed by the cross-national YouGov poll mentioned above (Abraham, 2018). Responses ranged on a five-point scale from not at all (1) to a great deal (5); the average is 2.96 and the standard deviation is 1.32. As documented by the YouGov survey (Abraham, 2018), respondents in France reported higher, on average, levels of feminist identity than respondents in other countries, but this average did not change over time. In other countries, there is a small increase in the averages for feminist identity in 2021 compared to other years (Figure 1).

### Political Interest

To measure people’s political interest, respondents were asked, “How interested would you say you are in politics?” Responses ranged on a four-point scale from not very interested (1) to very interested (4). In the pooled sample, the average level of political interest is 2.56 and the standard deviation is 0.97.

### Political Ideology

In order to measure people’s political ideology, we included the following information and question: “In politics, people sometimes talk of left and right. Where would you place YOURSELF on this scale?” Respondents recorded their answers on a scale ranging from left (0) to right (10). We recoded these responses so answers of 0–3 are identified as left-wing (21%) and answers of 7–10 (29%) are considered right-wing. The remaining people (50%) are considered moderates or have no affiliation. This approach to ideology follows earlier scholarship (Coffé & Bolzendahl, 2010; Durovic, 2017; Grasso, 2016; Suh & Reynolds-Stenson, 2018).

### Collective Efficacy

Respondents were asked to what degree they agree with the statement “Working as a group, people can influence government.” Respondents’ answers ranged from strongly disagree (1) to strongly agree (4). The average is 2.80 and the standard deviation is 0.82.

### Full-Time Employment Status

We asked respondents how they would best describe their employment situation. The values were recoded to include those who are employed full-time (1) and all other responses pooled together (0) (see also Coffé & Bolzendahl, 2017). Approximately 38% of respondents in our total sample of all four countries are employed full-time.

### Caregivers

Respondents were asked, “How many children under the age of 18 live in your home?” Responses ranged from no children to 8 or more children, but we recoded this variable into whether or not they had children (30% of respondents have children). We account for having children following scholarship in this area (Beauregard, 2016; Coffé & Bolzendahl, 2010, 2017; Elliott & Earl, 2018; Pfanzelt & Spies, 2019). This approach is also in line with Burciu and Hutter’s (2022) findings about differences based on caregiver roles. This approach is also consistent with work documenting how women with children (under the age of 18) in the home are more likely to participate in signing petitions, but less likely to vote (O’Neill & Gidengil, 2017). Self-identification as a feminist is slightly higher in 2021 for both caregivers and non-caregivers.

### Sex

**Figure 2.**
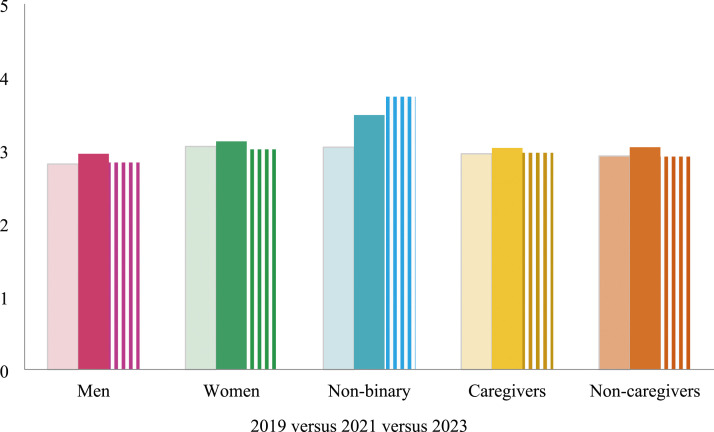
Feminist identity by year by gender and caregiving status.

The values of male, female, or non-binary were offered to respondents, to which only 88 chose non-binary (*n* = 18,362). This sample is too small to make conclusive claims, but the results are presented in hopes of inspiring further research about this group. The sample is composed of 49% male, 51% female, and 0.48% non-binary people. Following the cited scholarship, we translate these sex categories into gendered categories (men, women, and non-binary) when theorizing and interpreting results. Though the sample size is small, self-identification as a feminist is slightly higher, on average, among non-binary people and this average increases over time. For men and women, self-identification as a feminist is slightly higher in 2021 (Figure 2).

### Age

For age, we asked the year of birth then calculated age using the year of birth and year of data collection. Respondents were, on average, 48.31 years old (SD = 17.37).

### Education

For education, country-specific categories were recoded into four categories: high school diploma or less, some college, bachelor’s degree, and more than a bachelor’s degree. About half of the respondents (49%) identified as having high school or less, but there are cross-national variations in this portion. The average for the four-category variable is 1.93 and the standard deviation is 1.05 (see Table 1).

**Table 1.** Descriptive Statistics.

		Pooled sample	USA	UK	France	Canada
Women		51%	51%	50%	51%	52%
Non-binary		0.48%	0.53%	0.37%	0.24%	0.76%
Full-time work		38%	37%	37%	41%	38%
Caregiver (kids)		30%	31%	30%	32%	25%
Left-wing		21%	19%	19%	21%	23%
Right-wing		29%	37%	28%	28%	22%
Education (1–4)	Average	1.93	2.12	1.86	1.79	1.95
	SD	1.05	1.09	1.06	1.03	0.98
Age (18–100)	Average	48.31	48.06	47.91	48.82	48.45
	SD	17.37	17.96	17.24	16.86	17.37
Political interest (1–4)	Average	2.56	2.74	2.59	2.36	2.54
	SD	0.97	0.99	0.93	0.97	0.94
Collective efficacy (1–4)	Average	2.80	2.90	2.74	2.66	2.90
	SD	0.82	0.82	0.79	0.86	0.78
Signed online petition (1–4)	Average	1.84	1.79	1.96	1.86	1.77
	SD	1.00	1.01	1.02	0.99	0.96
Feminist identity (1–5)	Average	2.96	2.92	2.80	3.11	3.03
	SD	1.32	1.38	1.32	1.20	1.34

### Analysis

The analysis proceeds in a series of steps. First, we report a correlation matrix for our variables to examine the variables that influence both signing online petitions and feminist identity. Then, we analyze the role of feminist identity in predicting the frequency of signing online petitions using ordinary least squares regression (H1). Then, we run the models for each subgroup separately: men, women, and non-binary; caregivers versus non-caregivers, country, and year of data collection. We use figures to summarize the subgroup analysis, but the full regression models are in the Appendix.

### Ethics

The surveys received ethics approval (101662, 101856 and 102022) in accordance with Canada’s *Tri-Council Policy Statement: Ethical Conduct for Research Involving Humans.*

## Results

Before reviewing the multivariate results in relation to the hypotheses, Table 2 presents a correlation matrix of all variables. Feminist identity and signing online petitions are positively correlated (*r* = .217, *p* < .001). Those who identify as left-wing report higher levels of identification with feminism (*r* = .236, *p* < .001), whereas those who identify as right-wing report lower levels of feminist identity (*r* = −.065, *p* < .001). Women report slightly higher levels of identification with feminism (*r* = .072, *p* < .001) than men. Respondents from France report slightly higher levels (*r* = .065, *p* < .001) and respondents from the UK report lower levels of identification with feminism (*r* = −.073, *p* < .001) compared to respondents in other countries. Year of data collection does not relate to feminist identity, which means that feminist identity is not changing (uniformly) across time. As noted in relation to, feminist identity was slightly higher on average in 2021, compared to 2019 and 2023 in three of the four countries.

**Table 2.** Correlation Matrix.

		1	2	3	4	5	6	7	8	9	10	11	12	13	14	15	16
1 feminist identity	r	1															
	p																
2 online petitions	r	.217	1														
	p	<0.001															
3 efficacy	r	.260	.234	1													
	p	<0.001	<0.001														
4 interest	r	.225	.288	.385	1												
	p	<0.001	<0.001	<0.001													
5 age	r	−.048	−.128	.044	.123	1											
	p	<0.001	<0.001	<0.001	<0.001												
6 education	r	.167	.078	.140	.203	−.046	1										
	p	<0.001	<0.001	<0.001	<0.001	<0.001											
7 left-wing	r	.236	.111	.138	.133	−.025	.065	1									
	p	<0.001	<0.001	<0.001	<0.001	0.001	<0.001										
8 right-wing	r	−.065	.098	.053	.186	−.007	.070	−.325	1								
	p	<0.001	<0.001	<0.001	<0.001	0.336	<0.001	<0.001									
9 caregiver	r	.007	.118	.009	.009	−.370	.089	−.066	.080	1							
	p	0.345	<0.001	0.225	0.232	<0.001	<0.001	<0.001	<0.001								
10 full-time	r	.011	.042	.012	.029	−.291	.213	−.031	.078	.232	1						
	p	0.141	<0.001	0.096	<0.001	<0.001	<0.001	<0.001	<0.001	<0.001							
11 non-binary	r	.025	.018	.001	−.005	−.045	−.020	.031	−.018	.009	−.006	1					
	p	0.001	0.015	0.934	0.493	<0.001	0.008	<0.001	0.020	0.241	0.411						
12 women	r	.072	.007	−.046	−.192	−.125	−.033	.026	−.078	.051	−.129	−.071	1				
	p	<0.001	0.313	<0.001	<0.001	<0.001	<0.001	0.001	<0.001	<0.001	<0.001	<0.001					
13 US	r	−.021	−.030	.074	.107	−.008	.106	−.026	.106	.024	−.017	.004	.001	1			
	p	0.004	<0.001	<0.001	<0.001	0.250	<0.001	0.001	<0.001	0.001	0.025	0.545	0.900				
14 France	r	.065	.007	−.099	−.115	.017	−.078	.012	−.011	.027	.035	−.019	.002	−.335	1		
	p	<0.001	0.321	<0.001	<0.001	0.022	<0.001	0.123	0.165	<0.001	<0.001	0.008	0.810	<0.001			
15 UK	r	−.073	.066	−.045	.016	−.013	−.039	−.019	−.015	.004	−.014	−.009	−.016	−.336	−.327	1	
	p	<0.001	<0.001	<0.001	0.032	0.075	<0.001	0.013	0.059	0.599	0.067	0.237	0.035	<0.001	<0.001		
16 Canada	r	.029	−.043	.069	−.009	.005	.010	.034	−.083	−.055	−.005	.023	.013	−.340	−.330	−.332	1
	p	<0.001	<0.001	<0.001	0.208	0.517	0.189	<0.001	<0.001	<0.001	0.530	0.001	0.082	<0.001	<0.001	<0.001	
17 Year	r	−.001	−.014	−.018	−.028	−.007	−.008	−.009	.026	.047	.009	.006	.000	−.019	.009	.005	.005
	p	0.873	0.066	0.015	<0.001	0.313	0.264	0.244	0.001	<0.001	0.239	0.405	0.961	0.010	0.203	0.527	0.476

To assess the hypotheses, we conducted a multivariate regression analysis using ordinary least square regression analysis. Table 3 outlines the results for the pooled sample (H1). Controlling for gender and other factors, feminist identity is positively and significantly correlated with signing online petitions (*b* = 0.092, SE = 0.006, *p* < .001), as suggested in Hypothesis 1. Being a caregiver (*b* = 0.144, *SE* = 0.017, *p* < .001) and being a woman (*b* = 0.070, *SE* = 0.015, *p* < .001) are positively linked with signing online petitions in this multivariate model. Compared to US respondents (the reference group), respondents from the other three countries sign online petitions more often. We also note that collective efficacy (*b* = 0.155, *SE* = 0.010, *p* < .001) and political interest (*b* = 0.227, *SE* = 0.009, *p* < .001) are positively correlated with signing online petitions in this multivariate model. Identifying as left-wing (*b* = 0.160, *SE* = 0.020, *p* < .001) or as right-wing (*b* = 0.184, SE = .017, *p* < .001) are also correlated with signing online petitions. Older respondents sign online petitions less often than younger respondents (*b* = −0.008, *SE* = 0.001, *p* < .001). There are no significant differences in signing online petitions over time, as measured using the linear variable for the year of data collection.

**Table 3.** Ordinary Least Squares Regression for Signing Online Petitions.

	b	SE	B	p
Feminist self-identity	0.092	0.006	0.121	<0.001
Collective efficacy	0.155	0.010	0.123	<0.001
Political interest	0.227	0.009	0.214	<0.001
Age	−0.008	<0.001	−0.141	<0.001
Education	−0.009	0.007	−0.010	0.189
Left-wing (vs. Moderates)	0.160	0.020	0.064	<0.001
Right-wing (vs. Moderates)	0.184	0.017	0.083	<0.001
Caregivers (kids)	0.144	0.017	0.065	<0.001
Full-time	−0.044	0.016	−0.021	0.006
Non-binary	0.175	0.106	0.012	0.099
Women	0.070	0.015	0.035	<0.001
France (vs. US)	0.192	0.021	0.082	<0.001
UK (vs. US)	0.257	0.020	0.109	<0.001
Canada (vs. US)	0.040	0.020	0.017	0.051
Year of data collection (2019.2021.2023)	−0.007	0.004	−0.012	0.100

*n* = 16,620. Model R-squared = .163.

**Figure 3.**
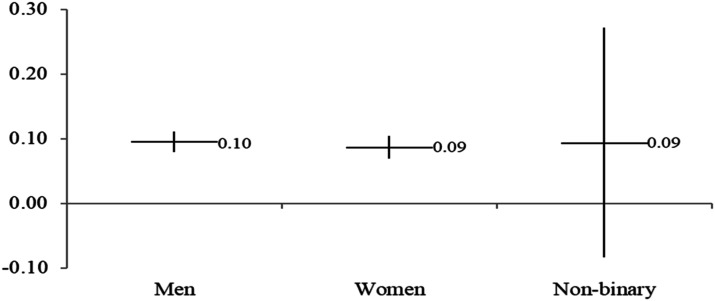
Feminist identity and online petitions for different genders.

**Figure 4.**
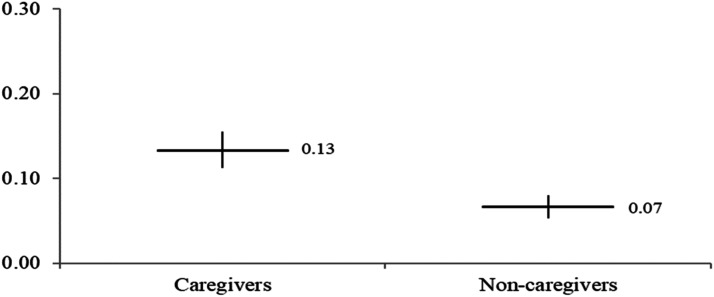
Feminist identity and online petitions by caregiver status.

**Figure 5.**
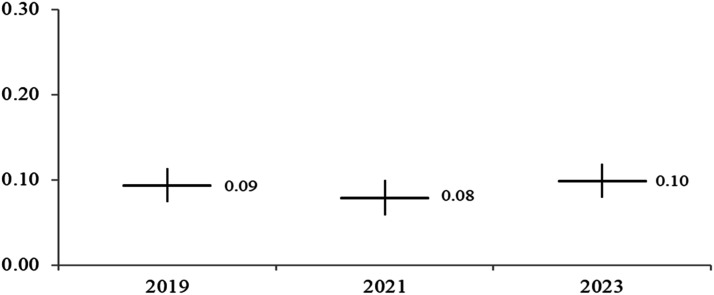
Feminist identity and online petitions by year.

**Figure 6.**
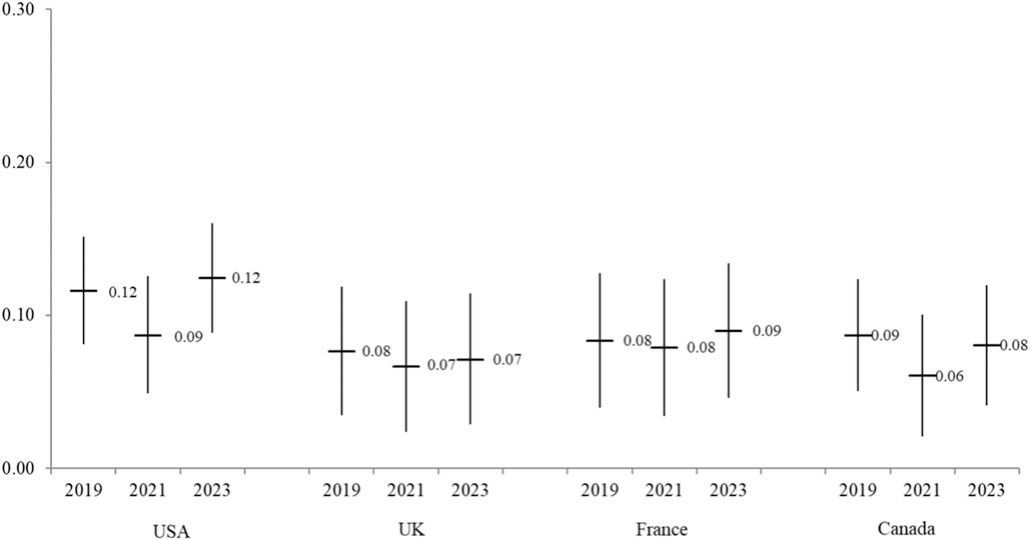
Feminist identity and online petitions by country and year.

Hypotheses 2 and 3 propose that feminist identity would have different mobilizing effects for different subgroups. This analysis produces 20 regression tables similar to Table 3. The full regression models are available in Appendix Tables 1 to 3 or Tables 4, 5, 6, and 7. To simplify the presentation of results, Figures 3, 4, 5, and 6 include the marginal effects for each subgroup. The unstandardized coefficients are provided with 95% confidence intervals. Confidence intervals that overlap suggest similar marginal effect estimates. In other words, an overlap in the intervals means no significant differences between the groups. Confidence intervals that overlap zero are considered to be not statistically significant at the .05 level.

**Table 4.** Ordinary Least Squares Regression for Signing Online Petitions for the US Sample.

	US				US				US			
	2019				2021				2023			
	b	SE	B	p	b	SE	B	p	b	SE	B	p
Feminist ID	0.116	0.018	0.162	<0.001	0.087	0.020	0.116	<0.001	0.124	0.018	0.168	<0.001
Collective efficacy	0.137	0.033	0.104	<0.001	0.170	0.031	0.140	<0.001	0.101	0.032	0.077	0.002
Political interest	0.277	0.027	0.265	<0.001	0.218	0.028	0.205	<0.001	0.286	0.028	0.274	<0.001
Age	−0.011	0.001	−0.196	<0.001	−0.013	0.002	−0.231	<0.001	−0.012	0.002	−0.206	<0.001
Education	−0.076	0.022	−0.083	0.001	−0.021	0.024	−0.022	0.401	0.028	0.024	0.030	0.246
Left-wing	0.069	0.064	0.028	0.285	0.264	0.071	0.100	<0.001	0.106	0.071	0.037	0.138
Right-wing	0.193	0.053	0.091	<0.001	0.252	0.056	0.118	<0.001	0.230	0.052	0.110	<0.001
Caregivers	0.171	0.056	0.074	0.002	0.212	0.062	0.093	0.001	0.280	0.055	0.136	<0.001
Full-time	0.026	0.052	0.013	0.613	0.038	0.057	0.018	0.503	0.074	0.055	0.035	0.180
Non-binary	−0.233	0.303	−0.017	0.443	0.007	0.325	0.001	0.982	0.010	0.326	0.001	0.975
Females	−0.016	0.047	−0.008	0.735	−0.037	0.050	−0.018	0.463	0.015	0.050	0.007	0.770
	*n* = 1579				*n* = 1397				*n* = 1396			
	Model R-squared = .228				Model R-squared = .234				Model R-squared = .301			

*The reference groups are moderates for ideology, other employment or no employment for employment status, and men for gender.

**Table 5.** Ordinary Least Squares Regression for Signing Online Petitions for the UK Sample.

	UK				UK				UK			
	2019				2021				2023			
	b	SE	B	p	b	SE	B	p	b	SE	B	p
Feminist ID	0.077	0.021	0.101	<0.001	0.067	0.022	0.085	0.002	0.071	0.022	0.091	0.001
Collective efficacy	0.177	0.038	0.133	<0.001	0.200	0.037	0.154	<0.001	0.162	0.037	0.117	<0.001
Political interest	0.190	0.033	0.169	<0.001	0.204	0.034	0.182	<0.001	0.273	0.033	0.238	<0.001
Age	−0.006	0.002	−0.106	<0.001	−0.008	0.002	−0.130	<0.001	−0.006	0.002	−0.099	0.001
Education	−0.026	0.026	−0.027	0.301	0.017	0.026	0.017	0.516	−0.008	0.026	−0.009	0.745
Left-wing	0.213	0.073	0.082	0.004	0.266	0.075	0.099	<0.001	0.285	0.072	0.111	<0.001
Right-wing	0.041	0.062	0.018	0.512	0.147	0.063	0.064	0.019	0.093	0.063	0.040	0.144
Caregivers	0.219	0.061	0.099	<0.001	0.111	0.064	0.048	0.083	0.136	0.062	0.060	0.030
Full-time	−0.100	0.058	−0.048	0.082	−0.108	0.058	−0.051	0.064	−0.014	0.059	−0.006	0.818
Non-binary	−0.911	0.673	−0.034	0.176	0.291	0.320	0.023	0.363	−0.027	0.555	−0.001	0.961
Females	0.129	0.058	0.064	0.027	0.188	0.055	0.091	<0.001	0.232	0.056	0.112	<0.001
	*n* = 1381				*n* = 1331				*n* = 1350			
	Model R-squared = .139				Model R-squared = .154				Model R-squared = .173			

*The reference groups are moderates for ideology, other employment or no employment for employment status, and men for gender.

**Table 6.** Ordinary Least Squares Regression for Signing Online Petitions for the France Sample.

	FR				FR				FR			
	2019				2021				2023			
	b	SE	B	p	b	SE	B	p	b	SE	B	p
Feminist ID	0.084	0.022	0.101	<0.001	0.079	0.023	0.094	<0.001	0.090	0.022	0.108	<0.001
Collective efficacy	0.236	0.032	0.199	<0.001	0.122	0.032	0.107	<0.001	0.137	0.033	0.114	<0.001
Political interest	0.171	0.030	0.162	<0.001	0.217	0.030	0.213	<0.001	0.237	0.030	0.229	<0.001
Age	−0.003	0.002	−0.055	0.056	−0.005	0.002	−0.074	0.012	−0.007	0.002	−0.119	<0.001
Education	−0.003	0.025	−0.004	0.893	−0.055	0.027	−0.056	0.040	−0.042	0.025	−0.044	0.098
Left-wing	0.251	0.066	0.102	<0.001	0.201	0.068	0.083	0.003	0.054	0.067	0.022	0.421
Right-wing	0.164	0.063	0.070	0.010	0.164	0.062	0.074	0.008	0.076	0.060	0.036	0.202
Caregivers	0.123	0.062	0.056	0.047	−0.057	0.061	−0.027	0.354	0.121	0.060	0.057	0.044
Full-time	−0.070	0.057	−0.034	0.224	−0.041	0.057	−0.020	0.472	−0.052	0.059	−0.026	0.375
Non-binary	1.106	0.670	0.041	0.099	−0.214	0.473	−0.012	0.651	0.105	0.419	0.006	0.803
Females	0.098	0.053	0.048	0.064	0.145	0.053	0.073	0.006	0.074	0.053	0.037	0.163
	*n* = 1397				*n* = 1353				*n* = 1360			
	Model R-squared = .144				Model R-squared = .107				Model R-squared = .124			

*The reference groups are moderates for ideology, other employment or no employment for employment status, and men for gender.

**Table 7.** Ordinary Least Squares Regression for Signing Online Petitions for the Canada Sample.

	CA				CA				CA			
	2019				2021				2023			
	b	SE	B	p	b	SE	B	p	b	SE	B	p
Feminist ID	0.087	0.019	0.127	<0.001	0.061	0.020	0.082	0.003	0.081	0.020	0.110	<0.001
Collective efficacy	0.161	0.036	0.123	<0.001	0.109	0.035	0.084	0.002	0.157	0.035	0.122	<0.001
Political interest	0.219	0.029	0.217	<0.001	0.213	0.032	0.193	<0.001	0.221	0.031	0.210	<0.001
Age	−0.005	0.002	−0.100	<0.001	−0.008	0.002	−0.146	<0.001	−0.009	0.002	−0.163	<0.001
Education	0.001	0.025	0.001	0.974	0.040	0.026	0.041	0.119	0.068	0.027	0.069	0.011
Left-wing	0.127	0.062	0.056	0.042	0.168	0.063	0.073	0.008	−0.027	0.065	−0.012	0.674
Right-wing	0.329	0.063	0.144	<0.001	0.182	0.065	0.076	0.005	0.308	0.065	0.134	<0.001
Caregivers	0.050	0.058	0.023	0.392	0.131	0.063	0.056	0.037	0.018	0.061	0.008	0.771
Full-time	−0.135	0.054	−0.070	0.012	−0.042	0.056	−0.020	0.453	−0.044	0.055	−0.022	0.421
Non-binary	0.864	0.329	0.066	0.009	0.382	0.378	0.026	0.312	0.468	0.238	0.051	0.049
Females	0.068	0.049	0.036	0.166	0.077	0.052	0.039	0.140	0.086	0.052	0.044	0.097
	*n* = 1363				*n* = 1396				*n* = 1317			
	Model R-squared = .166				Model R-squared = .126				Model R-squared = .181			

*The reference groups are moderates for ideology, other employment or no employment for employment status, and men for gender.

H2 proposed that feminist identity would have a greater role in women’s mobilization compared to men’s. However, we do not find significant differences for women (*b* = 0.087, *SE* = 0.009, *p* < .001) and men (*b* = 0.095, *SE* = 0.008, *p* < .001). These results, as well as those for the small sample of non-binary people, are presented in Figure 3 (full regression models are available in Appendix Table 1).

H3 proposes that caregivers would be mobilized more so than non-caregivers. We do find a significant difference (Figure 4). For caregivers, the relationship between having a feminist identity and signing petitions is larger (*b* = 0.134, *SE* = 0.011, *p* < .001) compared to non-caregivers (*b* = 0.067, *SE* = 0.007, *p* < .001). The coefficient is double the size for caregivers versus non-caregivers. As for gendered effects within the category of “caregivers,” Appendix Table 2 shows that women caregivers are not more likely to sign online petitions compared to caregivers who are men (*b* = 0.038, *SE* = 0.029, *p* = .186).

Having a feminist identity is not more mobilizing over time (Figure 5). The coefficients are similar (RQ2). In 2019, identifying as a feminist increases the frequency of signing petitions (b = 0.094, *SE* = 0.010, *p* < .001). The same relationship is observed for 2021 (*b* = 0.079, *SE* = 0.011, *p* < .001) and for 2023 (*b* = 0.099, *SE* = 0.010, *p* < .001). Across the three points in time, females sign petitions more frequently than males after accounting for the role of feminist identity, ideology, and other factors (Appendix Table 3).

In relation to RQ2, we find that feminist identity plays a similar role across time and countries (Figure 6). In other words, we do not find evidence of an increasing effect over time and for some countries versus others (RQ1). Instead, the role of feminist identity is consistent across time and country.

While our research questions focus on the role of feminist identity changing over time and by country, Tables 4 (US), 5 (UK), 6 (France), and 7 (Canada) also document other changes over time and offer more nuances about the findings. While we expected that 2021 would be an exceptional year with higher mobilization, we did not find this pattern in any country (Figure 6). In the US, we find a stronger positive correlation between left-wing ideology and online petitions in 2021 than in other years; since left-wing ideology correlates with feminist identity, the role of feminist identity is perhaps understated in the US context based on the modeling approach. This pattern is US-specific as we do not find it in other countries.

The relationship between caregiving and signing online petitions is stronger over time in the US, but in other countries, the pattern is different. In Canada, caregiving significantly correlates with signing online petitions in 2021, but not in other (non-pandemic) years. These findings are consistent with grievance theory in that the pandemic exacerbated inequality-motivated political participation. In contrast, in France and the UK, caregiving does not significantly correlate with signing online petitions in 2021 but does correlate with signing petitions in 2019 and 2023.

There are no significant differences between men and women in signing petitions in Canada and the US in any of the years of data collection in these models that consider feminist identity. However, in the UK, women are more likely to sign petitions online compared to men. In France, women are more likely to sign petitions in 2021, but the differences are not significant in other years.

## Discussion

Elliott and Earl (2018) argue that online petitions are one of the most popular and longstanding forms of online activism. This type of political engagement is particularly important as a low-cost option for marginalized groups (Elliott & Earl, 2018). Signing petitions is a popular political activity for women, which we explain in terms of their rights-based perspective on citizenship (Bolzendahl & Coffé, 2009). This activity became more important during the pandemic, as citizens faced lockdowns and restrictions in face-to-face activities, limiting opportunities to engage in other protest activities, such as marches and demonstrations. The pandemic also altered gender differences in care work and exacerbated grievances, which could lead to the development of a collective identity and subsequent participation in civic and political life to address these grievances. Women may resist the injustice of greater workloads within the home and participate in political activities to change this situation.

We find consistent patterns across the time periods: people who identified as feminists sign online petitions more often than people who do not identify as a feminist. We do not find large differences in or changes in the effects of having a feminist identity and signing petitions in 2023 compared to 2021 and 2019. Furthermore, while we find that women were slightly more likely to identify as a feminist, this collective identity does not have a stronger mobilizing effect for women than other groups. Instead, this collective identity is more mobilizing for caregivers, defined as those with children less than 18 years old, compared to non-caregivers. Holding a feminist identity is mobilizing for men and women caregivers. Our study is distinctive in understanding the different subgroups who could be mobilized, to a greater or lesser degree, by having a feminist identity.

We find that caregivers are more likely to be mobilized during this time period than non-caregivers. This finding highlights the importance of grievance-based approaches. The pandemic was a unique “critical event” (Ramos, 2008) that impacted most societies, whereas typically critical events encouraging activism occur at the local and national level. Due to this critical event, the average levels of feminist identity increase in 2021 compared to other years (see Figure 1); this higher level of feminist identity is observed in relation to men, women, caregivers, and non-caregivers. As such, these basic descriptive statistics suggest that this identity is indeed tied to grievances attached to the pandemic. However, this theory is not equally relevant across contexts. The findings suggest that grievance theory needs to attend to both country and year as contextual information to explain findings. Over time, the relationship between caregiving and signing online petitions becomes stronger in the US and caregiving is only significantly related to online petitions in Canada in 2021. Both findings offer support for grievance theory. However, in the multivariate models, feminist identity does not become more strongly related to online petitions in 2021 compared to the other years. Instead, the role of feminist identity on signing online petitions is consistent.

Our measures of political participation do not ask about the topic or issue attached to participation; it would be helpful to know if participants had signed a petition related to pandemic policies. For example, in the UK, three petitions were highly popular in the lead-up to survey data collection in February 2021. One petition requested that maternity leave be extended by three months due to the pandemic; the petition received more than 230,000 signatures. Another petition gathered more than 425,000 signatures, requesting that schools and colleges should be closed due to the increase in COVID-19 cases (https://petition.parliament.uk/petitions/550846). Another petition gathered more than 100,000 signatures requesting “a suspension of the duty on parents to secure regular school attendance for their child” (https://petition.parliament.uk/petitions/300399). Parents wanted the option to keep their children home from school without facing prosecution. Change.org documented more than 5000 petitions related to COVID-19 (https://www.change.org/t/covid-19-en-us) and many of these petitions were about opening or closing schools. These petitions had fewer signatures than the UK examples as most of them were directed at local or state decision-makers in the United States, reflecting the different government systems for the two countries.

Our theory discusses the pandemic as a critical global event that shaped grievances in four countries and we believe it has relevance for understanding more local and national experiences. For example, in the US context, women may be further mobilized by the repealing of Roe v. Wade (Kimport, 2022) in 2022, which has raised the salience of gender inequality in terms of reproductive rights. Beginning in September 2022, protestors took to the streets in Iran to protest the death of Mahsa Amini, who was arrested for not following the country’s dress code and later died in prison (Alkhaldi et al., 2022). This paper highlights gender differences related to caregiving that created grievances related to feminist identity; however, feminists may be mobilized by a variety of social issues. As such, we recommend further research to track changes in the salience of feminist identity in the mobilization process related to specific issues, such as reproductive and human rights.

Our study is distinctive in its cross-national approach. These four countries differ in terms of the willingness to identify as a feminist. We confirm Abraham’s (2018) finding that respondents from France report higher levels of identification with feminism and respondents from the UK report lower levels of identification with feminism, compared to respondents in other countries. While there are differences in adopting this identity, there are no differential effects on mobilization. Respondents from France who identify as being feminists are as likely to sign petitions as respondents from other countries who identify as being a feminist.

As noted, all four countries had income support in place in February 2021 (Hale et al., 2021). Additionally, during the month of February 2021, Canada and the United Kingdom had more school closures compared to the United States and France, which would imply differences in levels of grievances by country; the UK had the most stringent measures for the first year of the pandemic (Mathieu et al., 2020). However, we do not see country differences in the role of feminist identity on signing online petitions, despite expectations for differences in grievances.

While our sample is original in its cross-national design, the focus is on established Western democracies. Further research should consider a more diverse set of countries and greater diversity in the gender-based grievances in these countries, as the Iran example illustrates. Furthermore, we did not ask about race in survey data collection, as asking about race is particularly contentious in France. However, further research should consider racial, ethnic, and class differences within countries to better understand differences in collective identity and the implications on civic participation. In these four countries during these time periods, feminist identity is important for mobilizing civic participation, particularly for caregivers.

Beyond offering empirical evidence, this paper offers several theoretical contributions including understanding how collective identity combined with grievance theory relates to activism and theorizing both cross-national and longitudinal differences to offer a nuanced analysis of the role of context in political participation. We also offer new theoretical insights into the gendering of civic and political participation. While feminist identity is a significant predictor every year for every country, gender and caregiving status are much more idiosyncratic. To understand more global processes of mobilization, we need to consider variables that explain participation across a variety of contexts. Feminist identity does serve this role. Feminist identity can be mobilized “into meaningful and effective action” (Downing & Roush, 1984, p. 702). Our analysis looks beyond the mere categories of sex or gender into the social and political dynamics of how gender is constructed, leading to different roles; these roles can result in struggles that are politicized and translated into political behavior. We thus invite future research to engage with the ways in which identities are shaped and turned into mobilizing potential, particularly because we see that this potential goes further than the topic or condition that ignited identity-building in the first place.

## Appendix

Tables A1, A2 and A3

**Table A1.** Ordinary Least Squares Regression for Signing Online Petitions by Gender Group.

	Men				Women				Non-binary			
	b	SE	B	p	b	SE	B	p	b	SE	B	p
Feminist ID	0.095	0.008	0.129	<0.001	0.087	0.009	0.110	<0.001	0.094	0.089	0.132	0.294
Collective efficacy	0.159	0.014	0.124	<0.001	0.152	0.014	0.123	<0.001	−0.057	0.154	−0.050	0.713
Political interest	0.209	0.012	0.193	<0.001	0.242	0.012	0.226	<0.001	0.390	0.125	0.414	0.003
Age	−0.009	0.001	−0.147	<0.001	−0.008	0.001	−0.132	<0.001	−0.003	0.006	−0.053	0.647
Education	−0.005	0.010	−0.005	0.609	−0.020	0.011	−0.021	0.054	0.050	0.117	0.050	0.668
Left-wing	0.156	0.028	0.062	<0.001	0.171	0.028	0.070	<0.001	0.125	0.265	0.064	0.640
Right-wing	0.195	0.024	0.091	<0.001	0.167	0.026	0.072	<0.001	0.473	0.308	0.186	0.129
Caregivers	0.208	0.026	0.092	<0.001	0.087	0.024	0.040	<0.001	−0.137	0.216	−0.069	0.529
Full-time	−0.077	0.023	−0.038	<0.001	−0.028	0.023	−0.013	0.225	0.182	0.250	0.086	0.471
France	0.120	0.029	0.052	<0.001	0.269	0.029	0.115	<0.001	0.115	0.379	0.042	0.763
UK	0.154	0.028	0.067	<0.001	0.377	0.029	0.158	<0.001	0.206	0.309	0.083	0.508
Canada	−0.009	0.029	−0.004	0.761	0.087	0.029	0.037	0.002	0.442	0.269	0.223	0.105
Year	−0.009	0.006	−0.015	0.133	−0.007	0.006	−0.011	0.291	−0.043	0.068	−0.071	0.535

*The reference groups are moderates for ideology, other employment or no employment for employment status, and the US for country.

**Table A2.** Ordinary Least Squares Regression for Signing Online Petitions by Caregiver Group.

	Caregiver (kids)				Non-caregiver (no kids)			
	b	SE	B	P	b	SE	B	p
Feminist ID	0.134	0.011	0.166	<0.001	0.067	0.007	0.091	<0.001
Collective efficacy	0.144	0.018	0.115	<0.001	0.153	0.012	0.123	<0.001
Political interest	0.284	0.016	0.264	<0.001	0.198	0.010	0.191	<0.001
Age	−0.012	0.001	−0.130	<0.001	−0.008	0.001	−0.136	<0.001
Education	0.016	0.013	0.017	0.213	−0.027	0.009	−0.028	0.002
Left-wing	0.079	0.039	0.028	0.042	0.207	0.023	0.088	<0.001
Right-wing	0.265	0.031	0.121	<0.001	0.119	0.021	0.054	<0.001
Full-time employed	0.012	0.030	0.006	0.687	−0.079	0.020	−0.037	<0.001
Non-binary	0.056	0.176	0.004	0.752	0.275	0.132	0.018	0.037
Women	0.038	0.029	0.018	0.186	0.104	0.017	0.053	<0.001
France	0.031	0.037	0.013	0.411	0.284	0.025	0.124	<0.001
UK	0.176	0.037	0.072	<0.001	0.310	0.024	0.136	<0.001
Canada	−0.079	0.038	−0.031	0.039	0.103	0.024	0.046	<0.001
Year	0.001	0.008	0.002	0.902	−0.014	0.005	−0.023	0.007

*The reference groups are moderates for ideology, other employment or no employment for employment status, men for gender, and the US for country.

**Table A3.** Ordinary Least Squares Regression for Signing Online Petitions by Year.

	2019				2021				2023			
	b	SE	B	p	b	SE	B	p	b	SE	B	p
Feminist ID	0.094	0.010	0.126	<0.001	0.079	0.011	0.101	<0.001	0.099	0.010	0.128	<0.001
Collective efficacy	0.180	0.017	0.141	<0.001	0.151	0.017	0.124	<0.001	0.138	0.017	0.107	<0.001
Political interest	0.217	0.015	0.207	<0.001	0.212	0.015	0.199	<0.001	0.254	0.015	0.239	<0.001
Age	−0.007	0.001	−0.125	<0.001	−0.009	0.001	−0.150	<0.001	−0.009	0.001	−0.146	<0.001
Education	−0.030	0.012	−0.031	0.014	−0.007	0.013	−0.007	0.600	0.008	0.013	0.008	0.549
Left-wing	0.159	0.033	0.065	<0.001	0.222	0.034	0.089	<0.001	0.097	0.034	0.038	0.005
Right-wing	0.170	0.030	0.076	<0.001	0.201	0.031	0.090	<0.001	0.178	0.030	0.081	<0.001
Caregivers	0.151	0.030	0.068	<0.001	0.101	0.031	0.045	0.001	0.172	0.030	0.080	<0.001
Full-time	−0.076	0.027	−0.037	0.006	−0.049	0.028	−0.023	0.086	0.001	0.028	0.001	0.963
Non-binary	0.198	0.206	0.012	0.336	0.175	0.181	0.012	0.332	0.187	0.169	0.014	0.269
Females	0.056	0.026	0.028	0.028	0.086	0.026	0.043	<0.001	0.079	0.026	0.039	0.003
France	0.266	0.035	0.114	<0.001	0.236	0.037	0.101	<0.001	0.071	0.036	0.031	0.046
UK	0.240	0.034	0.103	<0.001	0.273	0.036	0.116	<0.001	0.258	0.035	0.110	<0.001
Canada	−0.032	0.034	−0.014	0.354	0.112	0.036	0.049	0.002	0.035	0.036	0.015	0.323

*The reference groups are moderates for ideology, other employment or no employment for employment status, men for gender, and the US for country.

## References

[bibr1-08944393241301050] Abendschön S. García-Albacete G. (2021). It’s a man’s (online) world: Personality traits and the gender gap in online political discussion. Information, Communication & Society, 24(14), 2054–2074. 10.1080/1369118X.2021.1962944

[bibr2-08944393241301050] Abraham T. (2018). When is a feminist not a feminist? YouGov. Retrieved from: https://yougov.co.uk/topics/politics/articles-reports/2018/03/08/when-feminist-not-feminist

[bibr3-08944393241301050] Ahn S. J. Cripe E. T. Foucault Welles B. McGregor S. C. Pearce K. E. Usher N. Vitak J. (2021). Academic caregivers on organizational and community resilience in academia (fuck individual resilience). Communication, Culture & Critique, 14(2), 301–305. 10.1093/ccc/tcab027

[bibr4-08944393241301050] Alkhaldi C. Pourahmadi A. John T. (2022). Clashes in Iran as thousands gather at Mahsa Amini’s grave, 40 days after her death. CNN. Retrieved from: https://www.cnn.com/2022/10/26/middleeast/iran-clashes-mahsa-amini-grave-intl/index.html

[bibr5-08944393241301050] Bargad A. Hyde J. S. (1991). Women’s studies: A study of feminist identity development in women. Psychology of Women Quarterly, 15(2), 181–201. 10.1111/j.1471-6402.1991.tb00791.x

[bibr6-08944393241301050] Beauregard K. (2016). At the intersection of gender and language: Why do Francophone women have lower levels of political participation? American Review of Canadian Studies, 46(1), 74–92. 10.1080/02722011.2016.1154882

[bibr7-08944393241301050] Bianchi S. M. Milkie M. A. Sayer L. C. Robinson J. P. (2000). Is anyone doing the housework? Trends in the gender division of household labor. Social Forces, 79(1), 191–228. 10.1093/sf/79.1.191

[bibr8-08944393241301050] Bianchi S. M. Sayer L. C. Milkie M. A. Robinson J. P. (2012). Housework: Who did, does or will do it, and how much does it matter? Social Forces, 91(1), 55–63. 10.1093/sf/sos120 PMC4242525 25429165

[bibr9-08944393241301050] Bode L. (2017). Closing the gap: Gender parity in political engagement on social media. Information, Communication & Society, 20(4), 587–603. 10.1080/1369118X.2016.1202302

[bibr10-08944393241301050] Bolzendahl C. Coffé H. (2009). Citizenship beyond politics: The importance of political, civil and social rights and responsibilities among women and men. British Journal of Sociology, 60(4), 763–791. 10.1111/j.1468-4446.2009.01274.x 19941492

[bibr11-08944393241301050] Boulianne S. (2023). Participatory inequality across countries: Contacting public officials online and offline. Social Science Computer Review, 41(4), 1336–1362. 10.1177/08944393211071067 37363157 PMC10285429

[bibr12-08944393241301050] Brundidge J. Baek K. Johnson T. J. Williams L. (2013). Does the medium still matter? The influence of gender and political connectedness on contacting U.S. Public officials online and offline. Sex Roles, 69(1-2), 3–15. 10.1007/s11199-013-0280-5

[bibr13-08944393241301050] Burciu R. Hutter S. (2022). More stress, less voice? The gender gap in political participation during the COVID-19 pandemic. European Journal of Politics and Gender, 6(1), 114–133. 10.1332/251510821X16602276230640

[bibr14-08944393241301050] Calarco J. M. Meanwell E. Anderson E. M. Knopf A. S. (2021). By default: How mothers in different-sex dual-earner couples account for inequalities in pandemic parenting. Socius: Sociological Research for a Dynamic World, 7(1), 1–15. 10.1177/23780231211038783

[bibr15-08944393241301050] Carreras M. (2018). Why no gender gap in electoral participation? A civic duty explanation. Electoral Studies, 52(1), 36–45. 10.1016/j.electstud.2018.01.007

[bibr16-08944393241301050] Coffé H. Bolzendahl C. (2010). Same game, different rules? Gender differences in political participation. Sex Roles, 62(1), 318–333. 10.1007/s11199-009-9729-y 20407575 PMC2852527

[bibr17-08944393241301050] Coffé H. Bolzendahl C. (2017). Avoiding the subject? Gender gaps in interpersonal political conflict avoidance and its consequences for political engagement. British Politics, 12(1), 135–156. 10.1057/bp.2016.9

[bibr18-08944393241301050] Cowan G. Mestlin M. Masek J. (1992). Predictors of feminist self-labeling. Sex Roles, 27(7-8), 321–330. 10.1007/bf00289942

[bibr19-08944393241301050] Crepaz M. M. L. Bodnaruk Jazayeri K. Polk J. (2016). What’s trust got to do with it? The effects of in-group and out-group trust on conventional and unconventional political participation. Social Science Quarterly, 98(1), 261–281. 10.1111/ssqu.12271

[bibr20-08944393241301050] Deckman M. McDonald J. Rouse S. Kromer M. (2020). Gen Z, gender, and COVID-19. Politics and Gender, 16(4), 1019–1027. 10.1017/S1743923X20000434

[bibr21-08944393241301050] Dodson K. (2015). Gendered activism: A cross-national view on gender differences in protest activity. Social Currents, 2(4), 377–392. 10.1177/2329496515603730

[bibr22-08944393241301050] Dodson K. (2016). TSMOs and protest participation. Socius: Sociological Research for a Dynamic World, 2(1), 1–14. 10.1177/2378023116680624

[bibr23-08944393241301050] Downing N. Roush K. (1984). From passive acceptance to active commitment: A model of feminist identity development for women. The Counseling Psychologist, 13(4), 695–709. 10.1177/0011000085134013

[bibr24-08944393241301050] Duncan L. E. (1999). Motivation for collective action: Group consciousness as mediator of personality, life experiences, and women’s rights activism. Political Psychology, 20(3), 611–635. 10.1111/0162-895X.00159

[bibr25-08944393241301050] Durovic A. (2017). A longitudinal analysis of gendered patterns in political action in France: A generational story? French Politics, 15(1), 418–442. 10.1057/s41253-017-0039-4

[bibr26-08944393241301050] Ejrnæs A. Harrebye S. F. (2021). How do crises paralyze and activate? The impact of dissatisfaction on European patterns of participation. European Politics and Society, 23(5), 597–616. 10.1080/23745118.2021.1911449

[bibr27-08944393241301050] Elliott T. Earl J. (2018). Online protest participation and the digital divide: Modeling the effect of the digital divide on online petition-signing. New Media & Society, 20(2), 698–719. 10.1177/1461444816669159

[bibr28-08944393241301050] Evans E. Chamberlain P. (2015). Critical waves: Exploring feminist identity, discourse and praxis in western feminism. Social Movement Studies, 14(4), 396–409. 10.1080/14742837.2014.964199

[bibr29-08944393241301050] Feezell J. T. (2016). Predicting online political participation: The importance of selection bias and selective exposure in the online setting. Political Research Quarterly, 69(3), 495–509. 10.1177/1065912916652503

[bibr31-08944393241301050] Giugni M. Grasso M. T. (2019). Party membership and social movement activism: A macro–micro analysis. Party Politics, 27(1), 92–102. 10.1177/1354068818823446

[bibr32-08944393241301050] Grasso M. T. (2016). Generations, political participation and social change in Western Europe. Routledge.

[bibr33-08944393241301050] Grasso M. T. Yoxon B. Karampampas S. Temple L. (2019). Relative deprivation and inequalities in social and political activism. Acta Politica, 54(66), 398–429. 10.1057/s41269-017-0072-y

[bibr34-08944393241301050] Ha S. E. Kim S. Jo S. H. (2013). Personality traits and political participation: Evidence from South Korea. Political Psychology, 34(4), 511–532. 10.1111/pops.12008

[bibr35-08944393241301050] Hale T. Angrist N. Goldszmidt R. Kira B. Petherick A. Phillips T. Webster S. , et al. (2021). A global panel database of pandemic policies (Oxford COVID-19 Government Response Tracker). Nature Human Behaviour, 5, 529–538. 10.1038/s41562-021-01079-8 33686204

[bibr36-08944393241301050] Haney T. J. Barber K. (2022). The extreme gendering of COVID−19: Household tasks and division of labour satisfaction during the pandemic. Canadian Review of Sociology/Revue canadienne de sociologie, 59(S1), 26–47. 10.1111/cars.12391 35946961 PMC9537987

[bibr37-08944393241301050] Heger K. Hoffmann C. P. (2021). Feminism! What is it good for? The role of feminism and political self-efficacy in women’s online political participation. Social Science Computer Review, 39(2), 226–244. 10.1177/0894439319865909

[bibr38-08944393241301050] Heger K. Hoffmann C. P. (2022). Feminist women’s online political participation: Empowerment through feminist political attitudes or feminist identity? Journal of Information Technology & Politics, 20(4), 1–14. 10.1080/19331681.2022.2119320

[bibr39-08944393241301050] hooks b. (1984). Feminist theory: From margin to center. South End Press.

[bibr40-08944393241301050] Hunt S. Benford R. A. (2004). Collective identity, solidarity, and commitment. In In Snow D. A. Soule S. A. Kriesi H. (Eds.), The Blackwell companion to social movements social movements (pp. 433–458). Retrieved from: 10.1002/9780470999103.ch19

[bibr41-08944393241301050] Kelly C. Kelly J. (1994). Who gets involved in collective action? Social psychological determinants of individual participation in trade unions. Human Relations, 47(1), 63–88. 10.1177/001872679404700104

[bibr42-08944393241301050] Kelly M. Gauchat G. (2016). Feminist identity, feminist politics: U.S. Feminists' attitudes toward social policies. Sociological Perspectives, 59(4), 855–872. 10.1177/0731121415594281

[bibr43-08944393241301050] Kimport K. (2022). Abortion after dobbs: Defendants, denials, and delays. Science Advances, 8(36), 1. 10.1126/sciadv.ade5327 PMC9451164 36070372

[bibr44-08944393241301050] Klandermans B. (2004). The demand and supply of participation: Social-psychological correlates of participation. In Snow D. A. Soule S. A. Kriesi H. (Eds.), The Blackwell companion to social movements social movements (pp. 360–379). Retrieved from: 10.1002/9780470999103.ch16

[bibr45-08944393241301050] Koc-Michalska K. Schiffrin A. Lopez A. Boulianne S. Bimber B. (2021). From online political posting to mansplaining: The gender gap and social media in political discussion. Social Science Computer Review, 39(2), 197–210. 10.1177/0894439319870259

[bibr46-08944393241301050] Lacombe M. J. Howat A. J. Rothschild J. E. (2019). Gun ownership as a social identity: Estimating behavioral and attitudinal relationships. Social Science Quarterly, 100(6), 2408–2424. 10.1111/ssqu.12710

[bibr47-08944393241301050] Li J. Giebler H. Wetter R. Lair H. K. Ellingwood J. (2023). Unequal electoral participation: The negative effects of long work hours and unsociable work schedules in Europe. European Journal of Politics and Gender, 6(1), 92–113. 10.1332/251510821X16602019188175

[bibr48-08944393241301050] Lorber J. (1997). The variety of feminisms and their contributions to gender equality. BIS Verlag.

[bibr49-08944393241301050] Mathieu E. Ritchie H. Rodés-Guirao L. Appel C. Gavrilov D. Giattino C. Hasell J. , et al. (2020). Coronavirus pandemic (COVID-19). In Our world in data. Retrieved from: https://ourworldindata.org/coronavirus

[bibr50-08944393241301050] McAfee N. Howard K. B. (2023). Feminist political philosophy. In Zalta E. N. Nodelman U. (Eds.), The Stanford encyclopedia of philosophy *(winter 2023 edition)*. Retrieved from: https://plato.stanford.edu/archives/win2023/entries/feminism-political

[bibr51-08944393241301050] Moseley M. W. (2018). Protest state: The rise of everyday contention in Latin America. Oxford University Press.

[bibr52-08944393241301050] Ohme J. Hameleers M. Brosius A. Van der Meer T. (2021). Attenuating the crisis: The relationship between media use, prosocial political participation, and holding misinformation beliefs during the COVID-19 pandemic. Journal of Elections, Public Opinion, and Parties, 31(sup1), 285–298. 10.1080/17457289.2021.1924735

[bibr53-08944393241301050] O’Neill B. Gidengil E. (2017). Motherhood’s role in shaping political and civic participation. In Thomas M. Bittner A. (Eds.), Mothers and others: The role of parenthood in politics (pp. 268–287).

[bibr54-08944393241301050] Oser J. Grinson A. Boulianne S. Halperin E. (2022). How political efficacy relates to online and offline political participation: A multi-level meta-analysis. Political Communication, 9(5), 607–633. 10.1080/10584609.2022.2086329

[bibr55-08944393241301050] Oser J. Hooghe M. Marien S. (2013). Is online participation distinct from offline participation? A latent class analysis of participation types and their stratification. Political Research Quarterly, 66(1), 91–101. 10.1177/1065912912436695

[bibr56-08944393241301050] Páez-Bernal C. Kittilson M. C. (2022). Gender and political participation. In Giugni M. Grasso M. T. (Eds.), The Oxford handbook of political participation (pp. 562–577). Oxford University Press.

[bibr57-08944393241301050] Pfanzelt H. Spies D. C. (2019). The gender gap in youth political participation: Evidence from Germany. Political Research Quarterly, 72(1), 34–48. 10.1177/1065912918775249

[bibr58-08944393241301050] Polletta F. Jasper J. M. (2001). Collective identity and social movements. Annual Review of Sociology, 27(1), 283–305. https://www.jstor.org/stable/2678623

[bibr59-08944393241301050] Ramos H. (2008). Opportunity for whom? Political opportunity and critical events in Canadian Aboriginal mobilization, 1951-2000. Social Forces, 87(2), 795–823. 10.1353/sof.0.0145

[bibr60-08944393241301050] Rhodebeck L. A. (1996). The structure of men’s and women’s feminist orientations: Feminist identity and feminist opinions. Gender & Society, 10(4), 368–403. 10.1177/089124396010004003

[bibr61-08944393241301050] Schwartz N. S. (2021). Guns in the north: Assessing the impact of social identity on firearms advocacy in Canada. Politics & Policy, 49(3), 795–818. 10.1111/polp.12412

[bibr62-08944393241301050] Siegel J. A. Calogero R. M. (2021). Measurement of feminist identity and attitudes over the past half century: A critical review and call for further research. Sex Roles, 85(5–6), 248–270. 10.1007/s11199-020-01219-w

[bibr63-08944393241301050] Snow D. (2001). Collective identity and expressive forms. Center for the Study of Democracy. https://escholarship.org/uc/item/2zn1t7bj

[bibr64-08944393241301050] Spelman E. V. (1990). Inessential woman. Beacon Press.

[bibr65-08944393241301050] Suh H. Reynolds-Stenson H. (2018). A contingent effect of trust? Interpersonal trust and social movement participation in political context. Social Science Quarterly, 99(4), 1484–1495. 10.1111/ssqu.12515

[bibr66-08944393241301050] Swank E. (2021). The gender conservatism of pro-life activists. Journal of Women, Politics & Policy, 42(2), 124–137. 10.1080/1554477X.2021.1842667

[bibr67-08944393241301050] Swank E. Fahs B. (2011). Pathways to political activism among Americans who have same-sex sexual contact. Sexuality Research and Social Policy, 8(1), 126–138. 10.1007/s13178-011-0034-5

[bibr68-08944393241301050] Swank E. Fahs B. (2014). Predictors of feminist activism among social work students in the United States. Social Work Education, 33(4), 519–532. 10.1080/02615479.2013.860096

[bibr69-08944393241301050] Swank E. Fahs B. (2017). Understanding feminist activism among women: Resources, consciousness, and social networks. Socius: Sociological Research for a Dynamic World, 3(1), 1–9. 10.1177/2378023117734081

[bibr70-08944393241301050] Szymanski D. M. (2004). Relations among dimensions of feminism and internalized heterosexism in lesbians and bisexual women. Sex Roles, 51(3/4), 145–159. 10.1023/B:SERS.0000037759.33014.55

[bibr71-08944393241301050] Tajfel H. (1978). Social categorization, social identity, and social comparison. In Tajfel H. (Ed.), Differentiation between social groups: Studies in the social psychology of intergroup relations (pp. 61–76). Academic Press.

[bibr72-08944393241301050] Veenstra K. Haslam S. A. (2000). Willingness to participate in industrial protest: Exploring social identification in context. British Journal of Social Psychology, 39(2), 153–172. 10.1348/014466600164390 10907093

[bibr73-08944393241301050] Wei L. (2012). Number matters: The multimodality of Internet use as an indicator of the digital inequalities. Journal of Computer-Mediated Communication, 17(3), 303–318. 10.1111/j.1083-6101.2012.01578.x

